# Does pre-existing cognitive impairment impact on amount of stroke rehabilitation received? An observational cohort study

**DOI:** 10.1177/0269215519843984

**Published:** 2019-04-25

**Authors:** Verity Longley, Sarah Peters, Caroline Swarbrick, Sarah Rhodes, Audrey Bowen

**Affiliations:** 1 Division of Neuroscience and Experimental Psychology, MAHSC, The University of Manchester, Manchester, UK; 2 CLAHRC Greater Manchester, Manchester, UK; 3 Manchester Centre for Health Psychology, MAHSC, The University of Manchester, Manchester, UK; 4 Division of Health Research, Lancaster University, Lancaster, UK; 5 Centre for Biostatistics, MAHSC, The University of Manchester, Manchester, UK

**Keywords:** Stroke, dementia, rehabilitation, cognitive impairment

## Abstract

**Objective:**

To examine whether stroke survivors in inpatient rehabilitation with pre-existing cognitive impairment receive less therapy than those without.

**Design:**

Prospective observational cohort.

**Setting:**

Four UK inpatient stroke rehabilitation units.

**Participants:**

A total of 139 stroke patients receiving rehabilitation, able to give informed consent/had an individual available to act as personal consultee. In total, 33 participants were categorized with pre-existing cognitive impairment based on routine documentation by clinicians and 106 without.

**Measures:**

Number of inpatient therapy sessions received during the first eight weeks post-stroke, referral to early supported discharge, and length of stay.

**Results:**

On average, participants with pre-existing cognitive impairment received 40 total physiotherapy and occupational therapy sessions compared to 56 for those without (mean difference = 16.0, 95% confidence interval (CI) = 2.9, 29.2), which was not fully explained by adjusting for potential confounders (age, sex, National Institutes of Health Stroke Scale (NIHSS), and pre-stroke modified Rankin Scale (mRS)). While those with pre-existing cognitive impairment received nine fewer single-discipline physiotherapy sessions (95% CI = 3.7, 14.8), they received similar amounts of single-discipline occupational therapy, psychology, and speech and language therapy; two more non-patient-facing occupational therapy sessions (95% CI = –4.3, –0.6); and nine fewer patient-facing occupational therapy sessions (95% CI = 3.5, 14.9). There was no evidence to suggest they were discharged earlier, but of the 85 participants discharged within eight weeks, 8 (42%) with pre-existing cognitive impairment were referred to early supported discharge compared to 47 (75%) without.

**Conclusion:**

People in stroke rehabilitation with pre-existing cognitive impairments receive less therapy than those without, but it remains unknown whether this affects outcomes.

## Introduction

Evidence suggests that people with a diagnosis of dementia prior to stroke are subject to a number of barriers around access to stroke rehabilitation.^1,2^ An estimated 10% of patients have a diagnosis of dementia prior to first stroke, and one-third of the patients develop dementia after recurrent stroke.^3^ Many patients may also have pre-clinical symptoms of dementia prior to a stroke.^4^ Pre-existing dementia is associated with higher levels of disability, risk of death, and likelihood of discharge to institutional care after stroke, when compared to patients without.^5–7^

In the United Kingdom, stroke services generally follow a pathway of hyper-acute/acute stroke care, inpatient rehabilitation, or early supported discharge (ESD) followed by community rehabilitation (although this varies depending on patient need and service organization). There is evidence to suggest that patients with dementia are able to benefit from ongoing stroke rehabilitation^8^ and no evidence to suggest they cannot.^9,10^ Despite this, people with pre-existing dementia are less likely to be referred or admitted for ongoing stroke rehabilitation than those without.^1,2,11,12^ Additional barriers to ongoing rehabilitation have been identified once admitted to post-acute services and include the time and priority that clinicians give patients with pre-existing dementia.^1,13,14^

No studies have described patients with pre-existing cognitive impairments who are seen by stroke services. Nor have studies examined whether patients with pre-existing dementia/cognitive impairment, who are deemed suitable for admission to ongoing inpatient rehabilitation, receive different amounts of ongoing stroke-specific rehabilitation than those without. The aim of this study was to examine whether undiagnosed pre-existing cognitive impairment or diagnosed dementia is associated with the amount of stroke-specific rehabilitation received in the inpatient rehabilitation phase, likelihood of transfer to ESD, and length of inpatient stay.

## Method

This prospective observational cohort study used clinical notes to extract data about pre-existing cognitive status and post-stroke rehabilitation received by participants. We obtained ethics and National Health Service (NHS) permissions (North West Haydock Research Ethics Committee 17/NW/0427) and used the Strengthening the Reporting of Observational Studies in Epidemiology (STROBE)^15^ checklist to report this study.

The study took place within four NHS stroke rehabilitation units in the United Kingdom supported by the UK National Institute for Health Research (NIHR) Clinical Research Network (CRN), who aided in identifying and approaching sites and participants. Eligible participants were inpatients on a stroke rehabilitation unit with a clinically confirmed stroke and under the care of the stroke team, capable of giving informed consent or had an individual available to act in the capacity of a personal/professional consultee, and identified by staff as having post-acute rehabilitation needs. Rehabilitation needs were evidenced by admission to the rehabilitation unit and confirmed by therapy staff as having active input at the time of consent rather than, for example, waiting for transfer to another setting. Ineligible patients were those considered to be in the last days of life, non-stroke, or unable to give informed consent and did not have an individual available/willing to act in the capacity of consultee. Staff taking consent followed the Mental Capacity Act (2005)^16^ principles and British Psychological Society guidelines^17^ when recruiting participants who lacked capacity to consent.

Consecutive sampling occurred across sites from August 2017 to January 2018. Local hospital research practitioners screened all patients on the stroke rehabilitation units, approached potentially eligible participants as soon as medically stable to receive information about the study, and gained informed consent where possible. We used standard and accessible/aphasia-friendly information sheets and consent forms designed using NIHR resources,^18^ alongside consultee declarations for participants deemed unable to give informed consent by hospital research practitioners.

We extracted data from consented participants’ clinical notes up to the first eight weeks post-stroke. Eight weeks was chosen to allow reasonable time for patients to receive rehabilitation services based on average length of stay from national data.^19^ The first author (V.L.) or hospital research practitioners accessed and manually reviewed clinical notes from hospital admission and counted every instance of documentation of an offered therapy session or activity by a therapist relating to the patient during the eight-week post-stroke data capture period. This was recorded on a paper case report form, and V.L. input these into a custom database. This data collection process was piloted with the first five recruits. We distinguished between patient-facing or non-patient-facing (i.e. phone calls, family meetings, etc.) activities and counted joint sessions as one of each of the present therapies.

We extracted data on pre-stroke cognitive functioning alongside routine demographic, clinical, and therapy data including number of routine post-stroke cognitive screens. Documented dementia diagnosis on admission or any details of documented evidence of pre-existence of cognitive impairment were noted. For example, if a participant had no recorded dementia diagnosis, but an occupational therapist documented a conversation with a relative who stated the patient was struggling with their memory, this was recorded as ‘pre-existing cognitive impairment’ from social history by an occupational therapist. If no dementia diagnosis or pre-existing cognitive impairment was documented, the patient was categorized as having no pre-existing cognitive impairment. Data extraction was not blind to cognitive status.

### Analysis

Participants were assigned to one of three groups during analysis based on data collected about their pre-stroke cognitive functioning. Those with a documented diagnosis of dementia on admission were assigned to the ‘dementia’ group. Remaining participants were then either categorized into the ‘pre-existing cognitive impairment’ group or the ‘no pre-existing cognitive impairment’ group. Post-stroke cognitive status did not inform analysis. No formal sample size calculation was used, as we knew nothing about variability of the size of effect to expect due to lack of previous studies in this population. A minimum of 20 participants per group and 20 participants per covariate were recommended in advance to ensure stable results from linear regression. Due to the small number of patients with diagnosed dementia, we carried out pre-planned aggregation of the dementia and pre-existing cognitive impairment groups. Combining these two groups was reasonable due to the fact that data were not available about severity of any pre-existing cognitive impairment; therefore, there was likely to be a large overlap in severity of cognitive impairment between these groups.

Statistical analyses were conducted using SPSS Statistics 23 for Windows. Our primary outcome measure was the total number of therapy sessions, calculated by combining total number of physiotherapy and occupational therapy sessions offered during the eight-week period. This was chosen because all patients on a rehabilitation ward typically receive these two therapies, whereas not all require speech and language therapy or psychological therapies.^20^ Discipline-specific sessions were also considered in secondary analyses (i.e. physiotherapy only), with further distinctions between patient-facing or non-patient-facing therapy sessions.

Descriptive characteristics by groups and the cohort are presented as total numbers and percentages for categorical variables and means and standard deviation (SD) and median for continuous variables. The primary outcome was examined with a linear regression adjusting for the possible confounders of age, sex, National Institutes of Health Stroke Scale^21^ (NIHSS; a standard measure of stroke severity), and pre-stroke modified Rankin Scale^22^ (mRS; a standard measure of functional disability) as extracted from admission records. Data were examined to clarify the distribution of residuals in order to meet the assumptions of linear regression. Missing data were handled using multiple imputation as sensitivity analysis using five imputed datasets in SPSS. Kaplan–Meier analysis and log rank (Mantel–Cox) test were used to test for differences between pre-existing cognitive impairment group and time until discharge (censored at eight weeks). Chi-squared (2 × 2) tests were used to test for differences between pre-existing cognitive impairment grouping and categorical referrals to ESD on discharge or not. The level of significance used was *p* < 0.05.

## Results

**Figure 1.**
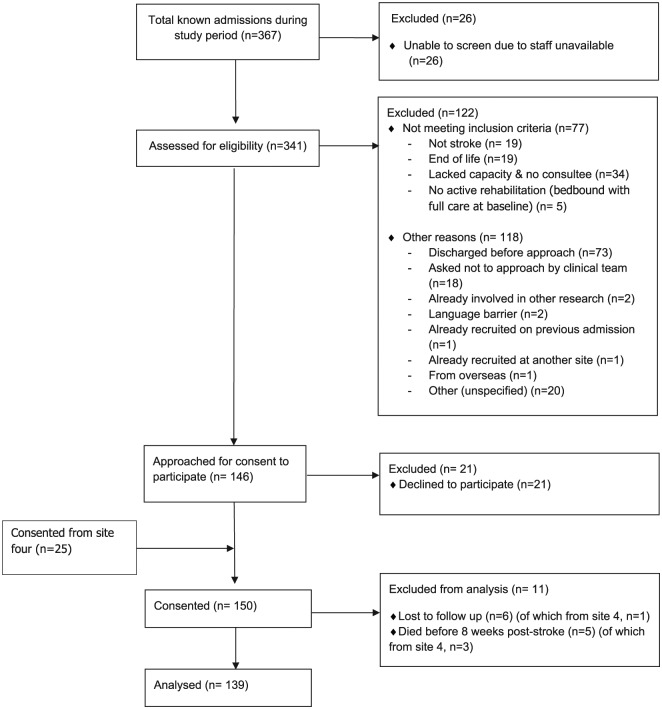
Flow diagram of available screening data.

We obtained complete screening data from three of four participating sites. Our screening data indicate that 52 (15%) patients on the wards were not approached due to lack of consultee or the clinical team advising not to approach. Consent rate for the three sites where it is known was 125 of 146 approached (86%; Figure 1). With the inclusion of 25 consenting participants from a fourth site that lacked screening data, a total of 139 of 150 consenting participants provided primary outcome data for analysis (attrition rate 7%).

Table 1 presents participant baseline demographic and clinical characteristics. The average age of participants was 75, with a median pre-stroke mRS of 0, which is similar to national audit figures.^19^ Median NIHSS on admission was 7, slightly higher than the national average.^19^ In total, 107 (77%) participants had a routine cognitive screen during admission to rehabilitation, most commonly with the Montreal Cognitive Assessment (MoCA).^23^

**Table 1.** Participant characteristics.

Characteristics	Total participants (*n* = 139), *N* (%)	No pre-existing cognitive impairment (*n* = 106), *N* (%)	Pre-existing cognitive impairment (n = 33)	
			Dementia (*n* = 9), *N* (%)	Undiagnosed pre-stroke cognitive impairment (*n* = 24), *N* (%)
Age, y, mean (SD)	75 (12.4)	73 (12)	83 (7.5)	83 (8.9)
Min and max	30–104	30–94	70–95	68–104
Female sex	83 (59.7)	64 (60.4)	3 (33.3)	16 (66.7)
Ethnicity				
White British	131 (94.2)	100 (94.3)	9 (100)	22 (91.7)
White Other	2 (1.4)	2 (1.9)	0	0
Mixed White and Black Caribbean	1 (0.7)	1 (0.9)	0	0
Asian or Asian British – any Asian background	5 (3.6)	3 (2.8)	0	2 (8.3)
Comorbidities				
Congestive heart failure	11 (7.9)	9 (8.5)	1 (11.1)	1 (4.2)
Hypertension	79 (56.8)	63 (59.4)	5 (55.6)	11 (45.8)
Diabetes	40 (28.8)	30 (28.3)	3 (33.3)	7 (29.2)
Previous stroke/TIA	37 (26.6)	25 (23.6)	2 (22.2)	10 (41.7)
Atrial fibrillation	26 (18.7)	19 (17.9)	2 (22.2)	5 (20.8)
Other neurological condition	3 (2.2)	3 (2.8)	0	0
Residential status on admission				
Living alone	48 (34.5)	33 (31.1)	2 (22.2)	13 (54.2)
Living with partner/others	81 (58.3)	67 (63.2)	4 (44.4)	10 (41.7)
Sheltered accommodation	8 (5.8)	5 (4.7)	2 (22.2)	1 (4.2)
Residential care	2 (1.4)	1 (0.9)	1 (11.1)	0
Pre-stroke mRS (*n* = 136)				
Mean (SD)	0.82 (1.3)	0.57 (1.1)	1.22 (1.3)	1.78 (1.4)
Median	0	0	1	2
NIHSS on admission (*n* = 121)				
Mean (SD)	8.56 (6.2)	8.78 (6.3)	7.89 (3.3)	7.95 (6.6)
Median	7	7.5	7	6
Days post-stroke on admission to rehabilitation unit				
Mean (SD)	5.83 (8.3)	6.19 (8.9)	2.67 (2.8)	5.46 (6.9)
Median	3	3	2	3
Days spent in rehabilitation unit (up to 56 days)				
Mean (SD)	38.42 (18.5)	39.07 (18.3)	33.78 (19.9)	37.33 (19.1)
Median	43	45	34	39.5
Cognitive screen completed, total screened (*n* = 107)				
MoCA	78 (72.9)	65 (74.7)	2 (40)	11 (73.3)
MMSE	3 (2.8)	3 (3.4)	0	0
Other	26 (24.3)	19 (21.8)	3 (60)	4 (79.3)

MMSE: Mini-Mental State Examination; TIA: transient ischemic attack; MoCA: Montreal Cognitive Assessment; mRS: modified Rankin Scale; NIHSS: National Institutes of Health Stroke Scale.

In total, 106 participants (76%) had no recorded pre-existing cognitive impairments, 9 (7%) had a diagnosis of dementia on admission, and 24 (17%) had documented pre-existing cognitive impairment. For the 24 participants with documented pre-existing cognitive impairment, the most common source of information was social history from family (*n* = 16, 67%). Occupational therapists were the professionals who most frequently documented existence of pre-existing cognitive impairment (*n* = 13, 54%), followed by physicians (*n* = 9, 38%), psychologists (*n* = 1, 4%), and mental health liaison staff (*n* = 1, 4%).

As planned, we combined the dementia and pre-existing cognitive impairment groups for analyses. All subsequent data are presented using two groups: no pre-existing cognitive impairment versus pre-existing cognitive impairment. Patients with pre-existing cognitive impairment had lower stroke severity (NIHSS mean difference = 1.8, 95% confidence interval (CI = 0.9, 2.8) and higher pre-stroke disability (mRS mean difference = 0.5, 95% CI = 0.6, 10.3) on average.

## Primary outcome

Participants with pre-existing cognitive impairment had on average 16 fewer total (physiotherapy and occupational therapy) sessions than participants with no pre-existing cognitive impairment (Table 2). This reduced to an average of 14 fewer sessions for participants with pre-existing cognitive impairment when analysis was adjusted for NIHSS, sex and age with both NIHSS (95% CI = 0.9, 2.8), and cognitive impairment grouping (95% CI = 0.5, 27.8) associated with number of sessions. Difference in number of sessions further reduced to an average of nine fewer therapy sessions for participants with pre-existing cognitive impairment when including pre-stroke mRS in adjusted analysis.

**Table 2.** Amount of therapy received by group.

	Number of therapy sessions received		
Type of therapy	No pre-existing cognitive impairment (*n* = 106) Mean (SD), median	Pre-existing cognitive impairment (*n* = 33) Mean (SD), median	Mean difference (95% confidence interval)
Total physiotherapy and occupational therapy	55.84 (35.3), 50	39.81 (25.5), 37	16.03 (2.89, 29.16) unadjusted
			14.1 (0.4, 27.8) adjusted^a^
			9.89 (–4.5, 24.2) adjusted^b^
Physiotherapy			
Patient facing	24.3 (16.2), 21.5	14.6 (10), 16	9.68 (3.7, 15)
Non-patient facing	3.59 (3.3), 3	4.03 (4.4), 2	−0.4 (–1.8, 0.9)
** **Total physiotherapy	27.91 (18.5), 24.5	18.66 (12.3), 18	9.24 (3.67, 14.82)
Occupational therapy			
Patient facing	22.8 (15.3), 20	13.6 (10.7), 10	9.21 (3.5, 14.9)
Non-patient facing	5.08 (4.3), 4	7.51 (5.8), 5	−2.4 (–4.3, –0.6)
** **Total occupational therapy	27.93 (18), 26.5	21.15 (14.9), 19	6.78 (–0.74, 13.63)
Speech and language therapy	9.14 (10.3), 5.5	7.64 (8), 5	1.5 (–2.39, 5.4)
Psychology	1.32 (2.8), 0	0.87 (1.7), 0	0.4 (–0.58, 1.47)

mRS: modified Rankin Scale; NIHSS: National Institutes of Health Stroke Scale.

a Adjusted to take into account sex, age, and NIHSS.

b Adjusted to take into account sex, age, NIHSS, and pre-stroke mRS.

Analyses were repeated using mean number of therapy sessions per week to account for differing lengths of stay between participants. Participants with pre-existing cognitive impairment had fewer sessions per week (mean difference = 1.7, 95% CI = 0.1, 3.4). NIHSS data were missing (not recorded in clinical notes) for 18 participants and assumed to be missing at random. Analysis using multiple imputation did not affect conclusions. Overall, participants with pre-existing cognitive impairments had fewer total therapy sessions and this was not fully explained by demographic and clinical variables.

## Secondary outcomes

When analysed by single discipline, participants with pre-existing cognitive impairment had fewer total physiotherapy sessions than those without pre-existing cognitive impairment (mean difference = 9.2, 95% CI = 3.7, 14.8). The differences in total occupational therapy, speech and language therapy, and psychology sessions were not statistically significant (Table 2). When examined by specific type of session, participants with pre-existing cognitive impairment had on average nine fewer patient-facing occupational therapy sessions (mean difference = 9.2, 95% CI = 3.5, 14.9) and on average two more non-patient-facing sessions than those without (mean difference = 2.4, 95% CI = 0.6, 4.3).

The median time to discharge from the rehabilitation units was 38 days for participants in the pre-existing cognitive impairment group compared to 45 days than those without. A log rank (Mantel–Cox) test revealed no differences in the time until discharge for the two groups (χ^2^(1) = 0.299, *p* = 0.585). In total, 54 participants were still inpatients at eight weeks. Of the 85 discharged by eight weeks, 47 (75%) participants without pre-existing cognitive impairment were referred to ESD compared to only 8 (42%) participants with pre-existing cognitive impairment, and this difference was significant (χ^2^(1) = 6.98, *p* = 0.008). In total, 54 (84%) participants without pre-existing cognitive impairment and 15 (71%) participants with pre-existing cognitive impairment were discharged to their previous residence. Similar proportions between groups were newly admitted to residential care; six (9%) without pre-existing cognitive impairment and two (10%) with. We attempted to use mRS to report outcome on discharge; however, because mRS is not routinely collected on admission, we were unable to calculate differences in outcome post-stroke.

## Discussion

We found that participants with documented pre-existing cognitive impairments, and who were deemed eligible for post-acute rehabilitation, received 16 fewer total therapy sessions over the first eight weeks post-stroke than participants without, and this was not fully explained by adjusting for potential confounders. These participants were also less likely to be referred to ESD. There was a small increase in amount of non-patient-facing occupational therapy received by participants with pre-existing cognitive impairments.

This is the first study to describe post-acute stroke rehabilitation for patients with pre-existing cognitive impairments; however, it has strengths and limitations. We demonstrated that it is possible to successfully recruit people with dementia/cognitive impairments to stroke research by using accessible consent procedures.^24,25^ In total, 42% of the total 150 consented participants were recruited using the consultee process, either because they were deemed to lack capacity or stroke-related communication impairments impacted on ability to consent. An additional strength is while the study took place in one region of the country, the four sites that participated varied widely in size and organization, adding to generalizability of the population and mediating potential bias.

Despite this, our screening data still demonstrate the difficulty of recruiting people with cognitive impairments to research, with 52 (15%) people not being approached about the study due to potential gatekeeping and lack of uptake of professional consultees. This is important considering the majority of our participants with pre-existing cognitive impairment/dementia 22 (66%) were recruited via the consultee process, indicating that some of those not approached due to lack of consultees may have had a pre-existing cognitive impairment. Equally, our study intentionally focused on those already admitted to rehabilitation, from which many stroke patients, including those with dementia, remain excluded.^10,12,26^ This may account for the relatively low level of dementia in our sample compared to the broader stroke population in the literature.^3,12^ We do not have data on the cognitive status of patients screened out of the study and therefore are unable to draw conclusions as to how many people with pre-existing cognitive impairment were excluded. Our findings may therefore in fact underestimate differences in amount of therapy received by patients across the whole stroke pathway.^26^

Further limitations may have been the use of existing data and inconsistent use of cognitive screening. This study relied on clinical documentation; some therapy input may have been undocumented; however, medical notes should contain all relevant information regarding care and reduce sources of bias.^27^ Some participants may have had pre-existing cognitive impairment that was not identified or documented by clinicians, and our data extractors were not blind to cognitive status of participants. Cognition was predominately screened using the MoCA; however, not all participants had a routine screen and post-stroke screening cannot detect pre-stroke ability. There are existing informant-based assessments of pre-existing cognition (such as the Informant Questionnaire on Cognitive Decline in the Elderly (IQCODE)),^28^ but none are validated in stroke populations and none were used in this study.^29^ Previous research highlights that social histories were considered to be a more important source of information than formal clinical assessment regarding pre-stroke cognition.^1^

Our findings reflect those of a number of studies which found that clinicians may question the value of stroke rehabilitation for patients with dementia, and this appears to include those with no formal dementia diagnosis.^1,11,12,30,31^ Factors such as stroke severity and previous level of independence have been found to be associated with quality of care after stroke.^32^ Our adjusted analysis supports this; stroke severity and previous level of disability were associated with amount of therapy provided. However, our measure of pre-stroke disability may be confounded by the existence of pre-existing cognitive impairment itself; mRS is a general measure, has no differentiation between physical or cognitive disability alone, and is prone to inter-observer variability, and so this adjusted analysis using mRS should be treated with caution.^22,33^ Similarly, only cardiovascular comorbidities were recorded; therefore, presence of other conditions that could impact on recovery are unknown in this sample.

An important point to note is whether the seeming inequality in amount of rehabilitation for people with pre-existing cognitive impairment (16 fewer therapy sessions across an eight-week period) is instead indicative of appropriate, less intensive, and personalized care. We are also unable to state whether this difference in therapy affected outcomes. A recent qualitative study found that clinicians stated they would provide shorter, more frequent sessions for people with pre-existing cognitive impairments,^1^ but our results do not find evidence of more frequent sessions and we did not collect data on session length. While therapists report a desire to provide multiple short interventions, provision of these types of sessions is rare.^34^ Our use of existing clinical data reduces potential subjectivity and adds validity to our findings.^35^ Equally, evidence suggests that patients with pre-existing cognitive impairments require longer time to make equivalent progress with rehabilitation than people without and that highly time-limited services are unable to meet these needs.^1^ We found a small increase in amount of non-patient-facing occupational therapy (i.e. phone calls, family meetings, etc.) received by participants with pre-existing cognitive impairments, which suggests that such patients might require different clinical resources in favour of more formal direct intervention. The lack of data on outcomes and severity of cognitive impairment for patients in this study limits the conclusions that can be drawn about the appropriateness of the overall difference in amount of therapy; however, the existence of such marked differences raises interesting questions that require further prospective investigation.

This study has a number of implications. We have demonstrated that there are a significant number of people within stroke rehabilitation services with undiagnosed pre-existing cognitive impairments, which is important given that pre-stroke cognitive decline is associated with future development of clinical dementia.^3^ We have also demonstrated that it is feasible to recruit patients with pre-existing cognitive impairments to stroke research. Our results suggest that a sizable group of stroke rehabilitation patients with pre-existing cognitive impairment receive less therapy. Increasing understanding of how to better identify this group is vital in order to ensure access to stroke rehabilitation and to enable rehabilitation to best meet the needs of patients.^36^ Stroke services need to reflect on the reasons for these differences.

Future research is required to examine long-term outcomes to see whether patients with pre-existing cognitive impairments, who we have shown receive less therapy, have worse outcomes and whether increasing the amount, duration, or type of therapy might counter this. Research is required to determine whether these observed differences in amount and type of rehabilitation are inequalities that need to be rebalanced or potentially reflect appropriate personalized care during the first eight weeks post-stroke.

**Clinical messages**

- A significant number of people within rehabilitation services were found to have had cognitive impairments prior to the stroke.
- People with pre-existing cognitive impairment who were deemed suitable for rehabilitation received 16 fewer therapy sessions over eight weeks than those without, especially physiotherapy.
